# Optimizing Sugarcane Clonal Propagation In Vitro by Using Calcium Ammonium Nitrate and Ammonium Sulfate

**DOI:** 10.3390/plants13192767

**Published:** 2024-10-02

**Authors:** Yuanli Wu, Faisal Mehdi, Zhengying Cao, Yimei Gan, Shuting Jiang, Limei Zan, Shuzhen Zhang, Benpeng Yang

**Affiliations:** 1 National Key Laboratory for Tropical Crop Breeding, Institute of Tropical Bioscience and Biotechnology, Chinese Academy of Tropical Agricultural Sciences, Haikou 571101, China; wuyuanli@itbb.org.cn (Y.W.); faisalmehdi@itbb.org.cn (F.M.); caozhengying@itbb.org.cn (Z.C.); ganyimei@itbb.org.cn (Y.G.); jiangshuting@itbb.org.cn (S.J.); zanlimei@itbb.org.cn (L.Z.); 2 Sanya Research Institute, Chinese Academy of Tropical Agricultural Sciences, Sanya 572024, China

**Keywords:** ammonium nitrate, potassium nitrate, calcium ammonium nitrate, ammonium sulfate, MS medium, sugarcane tissue culture

## Abstract

To replace explosive nitrate-based chemicals in MS medium, this study developed a new, safer, and more cost-effective method using fertilizer-grade calcium ammonium nitrate and ammonium sulfate. This approach replaces ammonium nitrate and potassium nitrate, ensuring both safety and cost efficiency for sugarcane propagation. Six local sugarcane varieties—Zhongtang1 (ZT1), Zhongtang3 (ZT3), Zhongtang6 (ZT6), Guitang42 (GT42), Guitang44 (GT44), and Guiliu 07150 (GT07150)—were used. In the control group (Ck), nitrate ions (NO_3_^−^) were 39.28 mM, and ammonium ions (NH_4_^+^) were 20.49 mM, with a 2:1 ratio. In the treatment groups, the concentrations of nitrate ions (NO_3_^−^) and ammonium ions (NH_4_^+^) included treatment 1 (19.69 mM NO_3_^−^ and 10.3 mM NH_4_^+^), treatment 2 (29.54 mM and 15.44 mM), treatment 3 (39.38 mM and 20.59 mM), treatment 4 (49.225 mM and 25.74 mM), treatment 5 (59.07 mM and 30.89 mM), and treatment 6 (68.915 mM and 36.03 mM), respectively, all with the same 2:1 ratio. Fifty bottles per treatment, with three replicates, were used for each sugarcane plantlets treatment. After five subcultures, the optimal ratio was determined by assessing morphological and physiological parameters, nitrogen levels, and SOD enzyme activity. The results indicated that treatment 3 (39.38 mM and 20.59 mM) and treatment 4 (49.225 mM and 25.74 mM) had the best morphological and physiological indicators. The optimal doses of calcium ammonium nitrate and ammonium sulfate were found in treatments 3 and 4, as well as in the control, with no significant difference among them. However, treatment 3, due to its lower dose, was more cost effective. To improve cost efficiency in practical production, it is recommended to use the lower concentration ratio of treatment 3 for plant tissue culture plantlets.

## 1. Introduction

Nitrogen is a crucial nutrient for plant growth and development. Ensuring healthy plant growth necessitates the provision of adequate nitrogen. Plants primarily absorb inorganic nitrogen in the form of nitrate ions (NO_3_^−^) and ammonium ions (NH_4_^+^). Relying on a single nitrogen source can result in low efficiency of nitrogen absorption and assimilation by plants [1,2]. Nitrates are considered dangerous and explosive in China, so there are strict controls on them. New rules, updated on 16 February 2011, require certificates for buying nitrates and proper qualifications for storing and transporting them. Additionally, companies handling nitrates must have the necessary storage and transportation qualifications. Given the low cost and low profit margins of nitrates, many reagent companies are reluctant to manage the complex certification process. Based on this, some manufacturers have introduced ready-to-use MS media, including both solid powder and liquid forms. However, the high cost of solid powder MS and liquid MS makes them unsuitable for large-scale factory farming. Moreover, the liquid medium has a short shelf life and is easy to precipitate calcium ions in long-term storage. Additionally, its organic components are susceptible to acidification, making it unsuitable for long-term storage. Consequently, liquid MS does not meet the requirements for applications in the sugarcane tissue culture industry. To address this issue, this study incorporated calcium ammonium nitrate (CAN) and ammonium sulfate as nutrient sources in the plant tissue culture medium. Additionally, both chemicals are highly soluble in water and are categorized as high-efficiency compound fertilizers. These fertilizers provide essential nitrogen in a readily available form and supply fast-acting calcium, making them particularly effective for promoting rapid nutrient uptake and supporting optimal plant growth. It provides a rapid fertilizer effect, quickly replenishing nitrogen to meet the needs for both ammonium nitrogen and nitrate nitrogen. Calcium ammonium nitrate is readily available in the market with a purity of up to 99%, making it suitable for use in culture medium preparations.

Supplying only nitrate ions will lead to the excessive accumulation of nitrate in plants, seriously affecting crop quality. Further, supplying only ammonium nitrogen in excess will cause ammonium toxicity and inhibit plant growth [3,4]. Generally, nitrate ions and ammonium nitrogen should be supplied simultaneously as nitrogen sources. Ammonium nitrogen can be directly utilized for amino acid synthesis. Numerous studies have demonstrated that providing both ammonium nitrogen and nitrate ions is beneficial to plants. For instance, an appropriate ratio of ammonium nitrogen to nitrate nitrogen can increase the reserve protein level in wheat grains and improve bread quality [5,6]. Additionally, it can enhance plant tolerance to salt and drought stress [7], improve plant responses to carbon dioxide [8,9], and increase the resistance of tomato plants to toxic *Pseudomonas* infection [10].

Factory breeding of healthy sugarcane plantlets is achieved through tissue culture. Generally, the nitrogen in the culture medium comes from potassium nitrate and ammonium nitrate. Potassium nitrate provides nitrate nitrogen, and ammonium nitrate provides both ammonium nitrogen and nitrate nitrogen. Crops have different preferences for ammonium nitrogen and nitrate nitrogen, but they have their own functions as plant nitrogen sources and cannot replace each other. In the actual factory-based breeding of healthy sugarcane plantlets, it is necessary to explore the requirements for the growth, proliferation, and rooting of sugarcane original plantlets using media produced with different proportions of ammonium nitrogen and nitrate nitrogen. It is important to understand the effects of different nitrogen forms and the ratio of ammonium nitrogen and nitrate nitrogen on plant morphology, growth, and physiology in order to provide a theoretical basis for the factory production of sugarcane plantlets.

Many studies have investigated the effects of nitrogen on crop physiology [10,11,12,13]. However, following the classification of nitrates as hazardous or explosive chemicals, there has been limited research on suitable substitutes in MS medium and the impact of different forms and ratios of nitrogen on the growth, physiological indices, ammonium and nitrate nitrogen levels, and SOD enzyme activity in sugarcane plantlets.

This study investigated the effects of different amounts of nitrate nitrogen and ammonium nitrogen at the same ratio on the plantlets of six sugarcane varieties using tissue culture methods. Therefore, the aim of study was to provide a theoretical basis for the rational use of nitrate substitutes and to improve their utilization rate for the tissue culture of sugarcane plantlets. This study explores the application of CAN and ammonium sulfate in the factory breeding of healthy sugarcane plantlets, addressing the issue of nitrogen deficiency in the culture medium due to the challenges in acquiring nitrates. In summary, this study aims to explore the application of calcium ammonium nitrate in the plant tissue culture industry to solve the problem of nitrogen deficiency in the culture medium caused by the difficulty in purchasing nitrates.

## 2. Materials and Methods

Plant material: for this study, six local cultivars—namely, Zhongtang1 (ZT1), Zhongtang3 (ZT3), Zhongtang6 (ZT6), Guitang42 (GT42), Guitang44 (GT44), and Guiliu 07150 (GT07150)—were used.

### 2.1. Experimental Methods

#### 2.1.1. Preparation of Culture Medium

In the original MS medium, nitrogen is provided by ammonium nitrate (NH_4_NO_3_) and potassium nitrate (KNO_3_). In this study, nitrate nitrogen and ammonium nitrogen were replaced by calcium ammonium nitrate [5Ca(NO_3_)_2_·NH_4_NO_3_·10H_2_O] and ammonium sulfate [(NH_4_)2SO_4_]. To better preserve the stock solution and maintain the balance of ion concentrations, potassium sulfate (K_2_SO_4_) and potassium chloride (KCl) were also added, each prepared as separate stock solutions. Stock solution 1 contained calcium ammonium nitrate, while stock solution 2 contained a mixture of ammonium sulfate, potassium sulfate, and potassium chloride. Stock solutions 1 and 2 were stored separately. The concentrations of components in stock solution 1 and stock solution 2 are shown in Table 1. The control group consisted of the original MS medium with ammonium nitrate and potassium nitrate, with the composition unchanged (Table 1). Under the condition of maintaining the ratio of ammonium nitrate to nitrate nitrogen in the MS medium, the amounts of these two components were gradually increased, setting up a total of 6 concentration gradients. Table 1 shows the ion concentrations in each treatment.

#### 2.1.2. Proliferation and Growth Rate of Sugarcane Plantlets

The original plantlets of the six aforementioned sugarcane varieties were inoculated into the culture medium of each treatment group. Fifty bottles were inoculated for each treatment, with three replicates per treatment, and sub-cultured every 15 days. The culture bottles used in the experiment had a volume of 200 mL, containing 30 mL of culture medium. Sucrose was used as the carbon source, with a concentration of 87.6 mM in both the multiplication and rooting media. Agar, at a concentration of 8 g/L, served as the solidifying agent, and the entire process was conducted using solid culture media. In the field, healthy first-year stems from each sugarcane variety were selected and cut into segments, ensuring that each segment contained at least one bud. The segments were then rinsed thoroughly with tap water to remove any surface contaminants. Subsequently, the stem segments were treated with distilled water at a constant temperature of 52 °C for 30 min to reduce microbial contamination. After the thermal treatment, the segments were planted in sterile coconut coir as a growth medium. The planted segments were placed in an artificial climate chamber maintained at a temperature of 38–40 °C for continuous heat treatment over a period of 8–10 days. During this time, the sugarcane axillary buds were monitored for growth, and when they reached a height of 10–15 cm, they were deemed suitable for use as explants in further experiments.

When the axillary buds reached a height of approximately 10–15 cm, the outer leaves were carefully removed. Under sterile conditions, the buds were disinfected by immersing them in 70% ethanol for 30 s, followed by immersion in a 10% sodium hypochlorite solution for 10 min. The buds were then rinsed 3–4 times with sterile water and dried using sterile filter paper. Next, 1–2 mm sections of the stem tip meristem were excised and inoculated onto the culture medium. This step was essential for the induction of multiple shoots.

Afterward, the multiplication stage was initiated. The small sugarcane shoots that grew on the initial medium were reinoculated onto the multiplication MS medium with a concentration of sucrose (87.6 mM) and 6-BA (4.44 μM), and the pH was adjusted to 5.8. They were cultured under a light intensity of 1000 lx with a photoperiod of 12 h per day. Subculturing was performed every 15 days, during which the MS medium was replaced with the specific medium used for each treatment. The multiplication plantlets in this experiment were those that had been sub-cultured five times. The cultures were maintained in an artificial climate chamber set to 28 °C, with a light intensity of 120 μmol m^−2^ s^−1^, under a controlled photoperiod of 16 h of light and 8 h of darkness per day. Relative humidity was consistently regulated between 55% and 60%. To strictly control the mutation rate, the number of subcultures was generally limited to within 10 generations. This experiment observed growth after five subcultures and analyzed the number of roots, root growth, and proliferation rate. The proliferation rate is calculated as follows:

$$Proliferation\;rate=\frac{\left(Number\;of\;shoots\;in\;the\;current\;generation-Number\;of\;shoots\;in\;the\;previous\;generation\right)}{\left(Number\;of\;shoots\;in\;the\;previous\;generation\right)}$$

#### 2.1.3. Rooting of Sugarcane Plantlets

For rooting, all plants were grown in rooting medium for each treatment. Before the plants were transferred to the rooting medium, all of the clustered multiplication shoots were unrooted. When they were transferred to the rooting medium, all the multiplication shoots from each treatment needed to be separated into individual plants, with each plant being inoculated into the rooting medium individually. The composition of the rooting medium was the same as the respective multiplication of MS medium for each treatment, with the addition of 0.00537 mM of naphthaleneacetic acid (NAA), and the pH was adjusted to 5.8. The specific procedure involved cutting the clustered multiplication shoots into individual plants and inoculating them onto the rooting medium with the concentrations of NAA (5.37 μM) and sucrose (87.6 mM). The plants were cultured under a light intensity of 1000–2000 lx with a photoperiod of 12 h per day. Here, the MS was replaced by the specific medium used for each treatment. The growth conditions were the same as above mentioned in Section 2.1.2. Fifty bottles were inoculated for each treatment, with three replicates. After 28 days of culture, the rooting conditions, including the number of roots and root length, were observed.

#### 2.1.4. Determination of Physiological Parameters of Sugarcane Plantlets

Chlorophyll was quantified employing the acetone extraction method [14]. Superoxide dismutase (SOD) activity was assessed via the NBT photochemical reduction method, as described by McCord [15]. Total soluble protein content was determined using the Coomassie Brilliant Blue G-250 method according to Bradford [16]. NH_4_^+^-N concentrations were measured using the modified ninhydrin method of Brandon [17], while NO_3_^−^-N levels were determined through the nitric acid reduction method [18].

### 2.2. Statistical Analysis

The data were analyzed and processed using DPS7.0 software. Significance analysis was performed using Duncan’s new multiple range method (*p* ≤ 0.05). Graphing was carried out using Excel 2007 software.

## 3. Results

**Figure 1:**
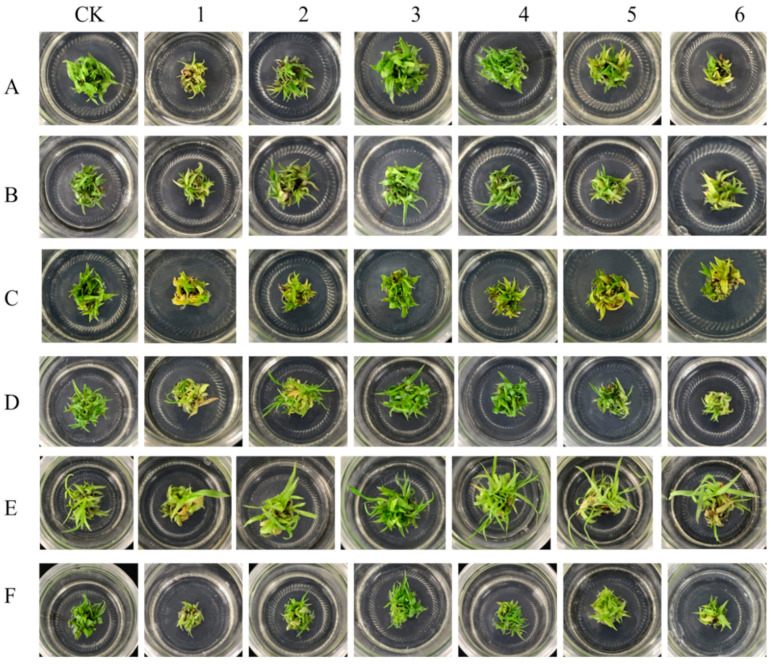
The growth response of six sugarcane plantlets varieties subjected to six different gradient ratios of ammonium nitrogen (NH_4_^+^-N) and nitrate nitrogen (NO_3_^−^-N). (**A**) GT 42, (**B**) GT 44, (**C**) GL 07150, (**D**) ZT 1, (**E**) ZT 3, and (**F**) ZT6. Note: CK (Control) contains ammonium nitrate and potassium nitrate.

The experimental results showed that at the same ratio, different amounts of ammonium nitrogen (NH_4_^+^-N) and nitrate nitrogen (NO_3_^−^-N) had significant effects on the growth status, leaf color, and proliferation rate of sugarcane plantlets. From treatment 1 to treatment 6, the six tested varieties exhibited a gradual increase in greenness and proliferation rate, with increasing ratios of NH_4_^+^-N to NO_3_^−^-N. Specifically, treatments 3 and 4 resulted in the darkest green leaf color and the most vigorous buds, showing no significant difference when compared to the control group (conventional MS culture medium) upon naked-eye observation. However, further increasing the NH_4_^+^-N to NO_3_^−^-N ratio led to a transition in leaf color from green to yellow and a corresponding decrease in proliferation rate. The following analysis is based on the detailed experimental results (Figure 1).

### 3.1. Effects on the Proliferation Rate of Sugarcane Plantlets

**Figure 2:**
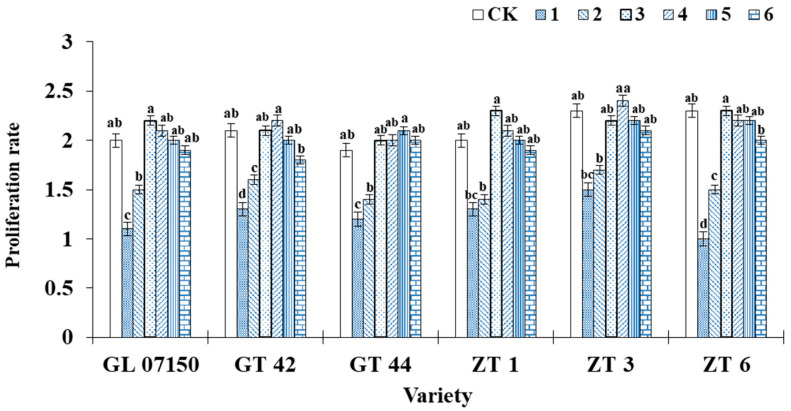
Illustrates the growth rate of sugarcane plantlets of six varieties under seven gradient ratios of ammonium nitrogen (NH_4_^+^-N) and nitrate nitrogen (NO_3_^−^-N). Different lowercase letters indicate significant differences (*p* < 0.05). Note: CK (Control) contains ammonium nitrate and potassium nitrate. Abbreviations: Zhongtang 1 (ZT1), Zhongtang 3 (ZT3), Zhongtang 6 (ZT6), Guitang 42 (GT142), Guitang 44 (GT144), and Guiliu 07150 (GL 07150).

The trend in the proliferation rate of original sugarcane plantlets as influenced by varying ratios of ammonium nitrogen (NH_4_^+^-N) to nitrate nitrogen (NO_3_^−^-N) is shown in Figure 2. The data indicated that the proliferation rate initially increased with the rising ratio, reaching a peak in treatments, and then subsequently declined. The maximum proliferation rates were observed in treatments 3 and 4, which showed no significant difference from the control group but which were significantly higher compared to treatments 1 and 2. Specifically, the varieties Guiliu 07150, Zhongtang 1, and Zhongtang 6 achieved their highest proliferation rates in treatment 3, while Zhongtang 3 exhibited the highest proliferation rate in treatment 4. From treatments 3–6, the proliferation rates of Guiliu 07150, Zhongtang 1, and Zhongtang 3 did not significantly differ from the control group. In contrast, Guiliu 42 and Zhongtang 6 showed a significant decrease in proliferation rate in treatment 6. Guitang 44 reached its maximum proliferation rate in treatment 5, with no significant difference from treatments 3, 4, 6, and the control group. Most tested varieties exhibited higher proliferation rates in treatments 3, 4, and 5. However, based on the growth conditions depicted in Figure 1, treatments 3 and 4 resulted in greener leaf color and stronger buds for each variety compared to treatment 5. This outcome is more conducive to later growth, expansion, and rooting quality requirements.

### 3.2. Impact on Sugarcane Plantlets Rooting

**Figure 3:**
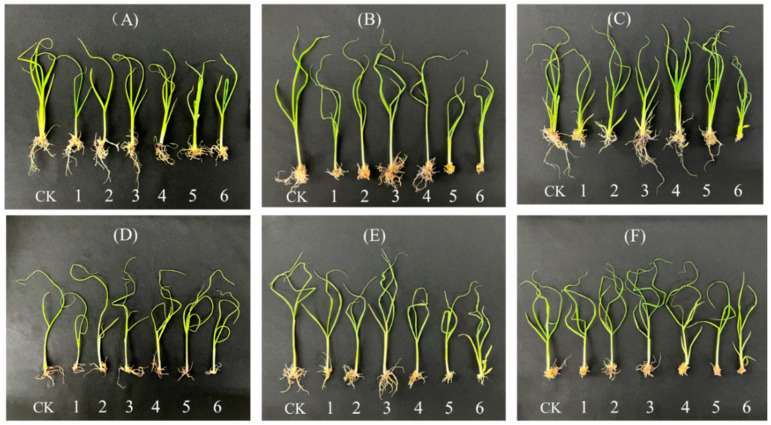
Root growth of six varieties of sugarcane original plantlets under six gradient ratios of ammonium nitrogen (NH_4_^+^-N) and nitrate nitrogen (NO_3_^−^-N). (**A**) GT 42, (**B**) GT 44, (**C**) GL 07150, (**D**) ZT 1, (**E**) ZT 3, and (**F**) ZT 6. Note: CK (Control) contains ammonium nitrate and potassium nitrate.

Under treatment 3, the root length and the number of hairy roots in ZT1 were visually superior compared to other treatments and showed no significant difference from the root growth observed in the MS medium (control). However, as the ratio of NH_4_^+^ to NO_3_^−^ continued to rise, both the root length and the number of hairy roots exhibited a noticeable decline (Figure 3).

**Figure 4:**
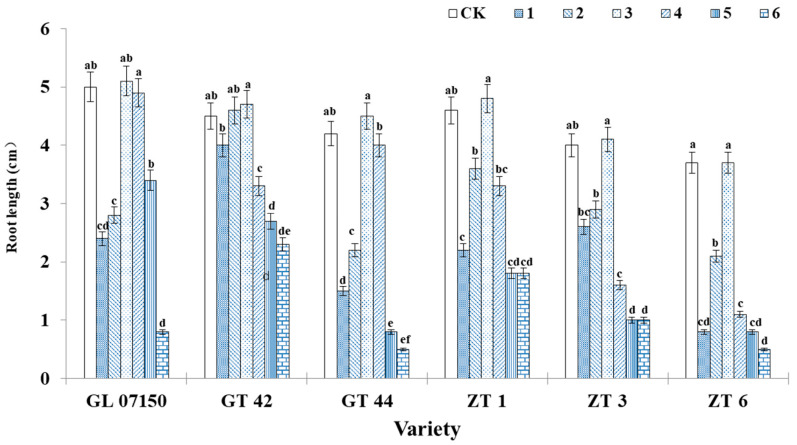
The average root length of six sugarcane original plantlets under seven gradient ratios of ammonium nitrogen (NH_4_^+^-N) and nitrate nitrogen (NO_3_^−^-N). Different lowercase letters indicate significant differences (*p* < 0.05). Note: CK (Control) contains ammonium nitrate and potassium nitrate.

**Figure 5:**
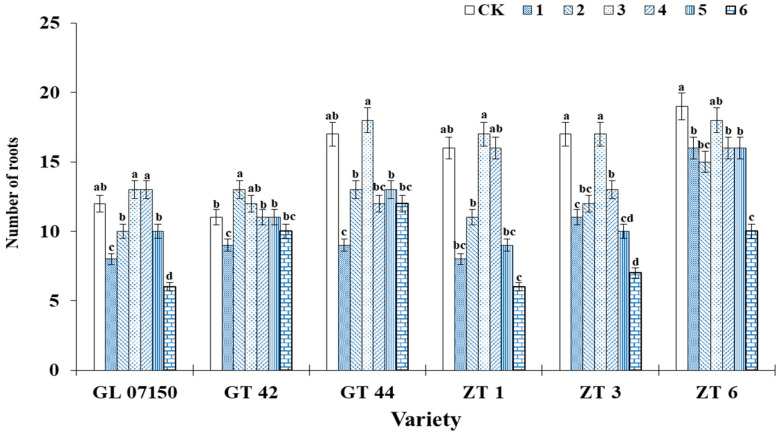
This figure illustrates the average number of roots in six sugarcane original plantlets under seven gradient ratios of ammonium nitrogen (NH_4_^+^-N) and nitrate nitrogen (NO_3_^−^-N). Different lowercase letters indicate significant differences (*p* < 0.05). Note: CK (Control) contains ammonium nitrate and potassium nitrate.

As observed in Figure 4, under treatment 3, the average root length growth of the six tested sugarcane varieties was optimal, achieving the longest root elongation with no significant difference from the control group. The root elongation of GT44, ZT 1, ZT3, and ZT6 under treatment 3 was significantly higher than that observed in other treatment groups. Regarding the number of hairy roots, the trends in root length changes for each variety were similar. Under treatment 3, most varieties exhibited the highest number of hairy roots, as depicted in Figure 5. For the four varieties G07150 (with equal root numbers in treatments 3 and 4), GT44, ZT1, and ZT3, there were no significant differences between treatment 3 and the control group, or they were the same, and these values were significantly higher than those in other treatment groups. The number of roots for GT42 was maximized under treatment 2, which was not significantly different from treatment 3 but was significantly higher than the control group and other treatment groups. ZT6 exhibited the largest number of roots in the control group, which was not significantly different from treatment 3 and significantly higher than in other treatment groups. In summary, treatment 3 optimizes the root elongation and the number of hairy roots for each tested variety, indicating a more favorable growth condition (Figure 5).

### 3.3. SOD Activity

**Figure 6:**
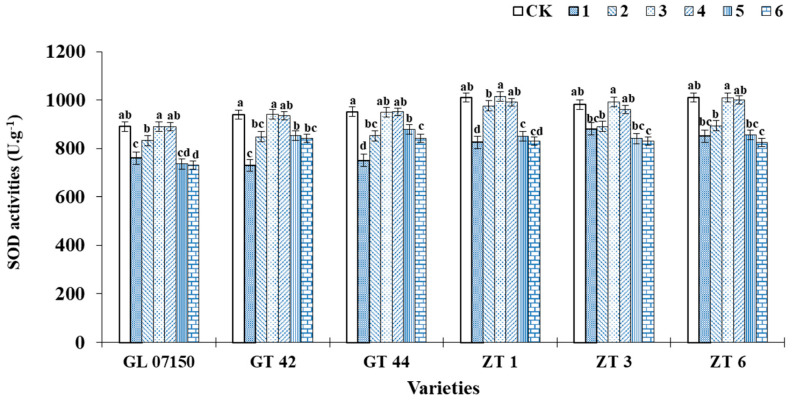
This figure represents the SOD activity of six sugarcane original plantlets under seven gradient ratios of ammonium nitrogen (NH_4_^+^-N) and nitrate nitrogen (NO_3_^−^-N). Different lowercase letters indicate significant differences (*p* < 0.05). Note: CK (Control) contains ammonium nitrate and potassium nitrate.

In this experiment, as the ratio of NH_4_^+^ to NO_3_^−^ in the culture medium increased, the SOD activity of the six varieties of sugarcane plantlets initially increased and then decreased. Under treatments 3 and 4, the SOD activity of each variety reached its peak. As depicted in Figure 6, the superoxide dismutase (SOD) activity of each tested variety reached its highest levels under treatments 3 and 4. The SOD activity of G07150, ZT1, ZT3, and ZT6 peaked under treatment 3, with no significant difference from the control group, and treatment 4, but was significantly higher than in other treatment groups. The SOD activity of GT42 under treatment 3 was consistent with the control group, showing no significant difference from treatment 4, but was superior to other treatment groups. The control group of GT44 exhibited the highest SOD activity; however, the difference in SOD activity compared to treatments 3 and 4 was not significant. Therefore, under treatments 3 and 4, the SOD activity of the sugarcane plantlets of the six tested varieties was higher, indicating a lower degree of oxidative stress Figure 6).

### 3.4. Chlorophyll Determination

**Figure 7:**
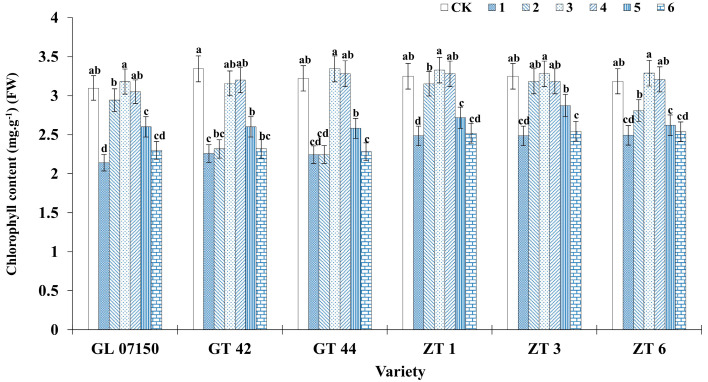
Chlorophyll content of six sugarcane original plantlets under seven gradient ratios of ammonium nitrogen (NH_4_^+^-N) and nitrate nitrogen (NO_3_^−^-N). Different lowercase letters indicate significant differences (*p* < 0.05). Note: CK (Control) contains ammonium nitrate and potassium nitrate.

Different nitrogen form ratios have varying effects on the chlorophyll content of leaves in sugarcane plantlets. As the ratio of NH_4_^+^ to NO_3_^−^ increased across treatments, the total chlorophyll content of leaves from the six sugarcane varieties initially increased and then decreased. The peak chlorophyll content was observed in treatments 3 and 4, as illustrated in Figure 7. For the varieties G 07150, GT44, ZT1, ZT3, and ZT6, the maximum chlorophyll content was recorded under treatment 3, with no significant difference compared to the control group and treatment 4. Specifically, for ZT3, the chlorophyll content under treatment 3 was similar to that under treatment 2 and significantly higher than in other treatment groups. GT42 showed the highest chlorophyll content in the control group, with no significant difference from treatments 3 and 4. The analysis of Figure 1 and Figure 7 shows that as the ratio of NH_4_^+^ and NO_3_^−^ increased, the leaf color of various sugarcane varieties gradually changed from yellow to green. When the ratio concentration of treatments 3 and 4 was reached, the leaf color became particularly dark green. Compared with the control group in MS medium, the difference in leaf color observed with the naked eye was not significant. However, as the dosage and ratio of NH_4_^+^ and NO_3_^−^ further increased, the leaf color obviously changed from green to yellow. This phenomenon shows that the synthesis of chlorophyll is promoted under the ratio of NH_4_^+^ and NO_3_^−^ in treatments 3 and 4.

### 3.5. Soluble Protein Quantification

**Figure 8:**
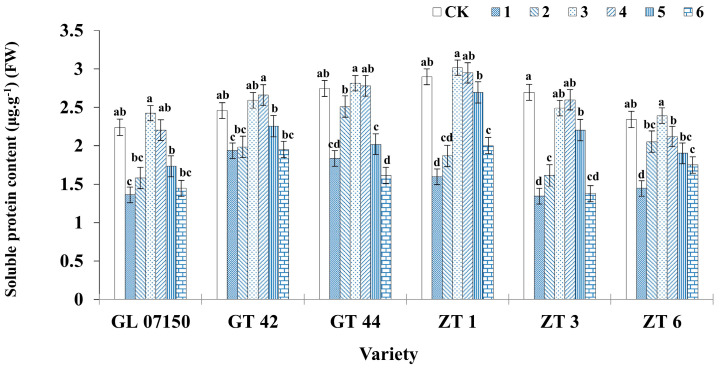
Soluble protein content in six sugarcane plantlets under seven gradient ratios of ammonium nitrogen (NH_4_^+^-N) and nitrate nitrogen (NO_3_^−^-N). Different lowercase letters indicate significant differences (*p* < 0.05). Note: CK (Control) contains ammonium nitrate and potassium nitrate.

As the dosage of NH_4_^+^ and NO_3_^−^ in the culture medium increased from a ratio of 20:20 to 50:50, the soluble protein content in each variety of sugarcane plantlet exhibited a gradual increase. However, further increases in the ratio of NH_4_^+^ to NO_3_^−^ led to a significant decrease in the soluble protein content in most of the tested varieties. During the entire treatment period, the soluble protein content in the six varieties of sugarcane plantlets initially increased and then decreased, peaking during treatments 3 and 4. In treatment 3, the soluble protein concentration of G07150, ZT1, and ZT6 was higher than that of the control group. There was no significant difference compared to treatment 4, but these values were significantly higher than those in other treatment groups, with ZT1 showing a particularly marked increase. The soluble protein content of GT42 in treatments 3 and 4 was also higher than in the control group, although the difference was not significant. ZT3 maintained a higher soluble protein concentration in the original MS medium but did not show a significant difference from treatments 3 and 4. In summary, the six varieties achieved high soluble protein concentrations under both treatments 3 and 4, with treatment 3 showing a slightly better effect compared to treatment 4 (Figure 8).

### 3.6. Determination of Nitrogen (Ammonium Nitrogen and Nitrate Nitrogen)

**Figure 9:**
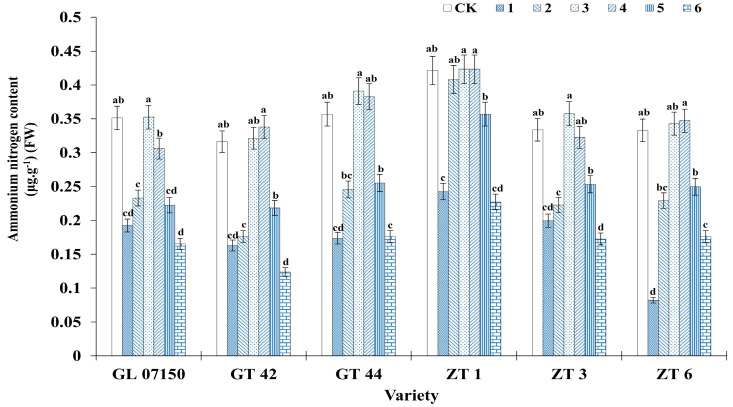
Ammonium nitrogen content in six sugarcane original plantlets under seven gradient ratios of ammonium nitrogen (NH_4_^+^-N) and nitrate nitrogen (NO_3_^−^-N). Different lowercase letters indicate significant differences (*p* < 0.05). Note: CK (Control) contains ammonium nitrate and potassium nitrate.

**Figure 10:**
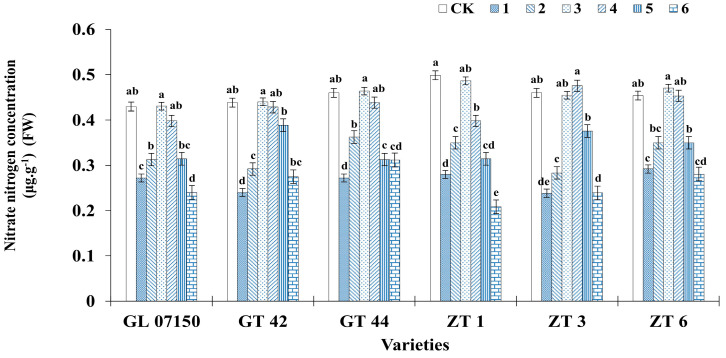
Nitrate nitrogen content in six sugarcane original plantlets under seven gradient ratios of ammonium nitrogen (NH_4_^+^-N) and nitrate nitrogen (NO_3_^−^-N). Different lowercase letters indicate significant differences (*p* < 0.05). Note: CK (Control) contains ammonium nitrate and potassium nitrate.

Figure 9 and Figure 10 clearly illustrate the significant impact of varying nitrogen form ratios on the nitrogen content in sugarcane. Analysis of the ammonium nitrogen and nitrate nitrogen content across the six varieties of sugarcane plantlets indicates a synergistic effect in the absorption of both nitrogen forms. As the ratio of ammonium nitrogen to nitrate nitrogen in the culture medium increased, the content of both nitrogen forms initially increased and then decreased, with peak contents observed in treatments 3 and 4. For ammonium nitrogen, there was no significant difference between the sugarcane plantlets under treatments 3 and 4 compared to the control group for the six tested varieties. Specifically, the ammonium nitrogen content in four varieties G07150, GT44, ZT1 (with equivalent content in treatments 3 and 4), and ZT3 was higher than in the conventional MS medium control, though the difference was not significant. GT 42 and ZT6 exhibited peak ammonium nitrogen content under treatment 4, with no significant difference from their respective control groups and treatment 3 (see Figure 9). Similarly, the nitrate nitrogen content for the six varieties peaked under treatments 3 and 4. Four varieties—G07150, GT 42, GT 44, and ZT6—had nitrate nitrogen content exceeding that of the conventional MS medium control, but the differences were not significant. ZT3 showed maximum nitrate nitrogen content under treatment 4, with no significant difference from the control group or treatment 3. ZT1 had the highest nitrate nitrogen content in the control group, with no significant difference compared to treatment 3, though a significant reduction was observed in treatment 4. For the remaining five varieties, there were no significant differences in nitrate nitrogen content among treatments 3, 4, and the control group, with levels significantly higher than in other treatment groups (see Figure 10).

Cost Analysis of MS Media Nutrients: Considering the need to control production costs, using the preparation of 100 L of MS medium as an example, the cost of the original MS formulation is 449.7 RMB (all amounts are in Chinese Yuan), while the commercially prepared solid powdered MS medium costs 712.04 RMB, and the commercially prepared liquid MS medium costs 1503 RMB. The cost of the formulation for treatment 3 is 423.14 RMB, and the cost for treatment 4 is 425.37 RMB (see Table 2 and Table 3).

## 4. Discussion

The quality of plant growth is directly reflected in its growth state and biomass. This can serve as a solid basis for evaluating the effects of different ammonium nitrogen and nitrate nitrogen ratios on the growth and metabolic activities of sugarcane plantlets. Nitrogen and its form ratios directly affect the life activities of plants, which are ultimately manifested in the external growth form and biomass of the plants [19]. Additionally, some researchers have pointed out that MS medium was initially developed for the rapid growth of tobacco callus, rather than focusing on plant regeneration and proliferation. This formulation makes chloride the main anion after nitrate, with a relatively low phosphorus content. The ability of chloride to induce rapid cell division allows for the swift growth of callus tissue [13]. However, MS medium remains widely used in most plant tissue cultures as it provides a comprehensive array of nutrients. There is still significant demand for it in the factory propagation of sugarcane. Therefore, in this experiment, we adjusted the ion concentrations when using readily available chemicals such as calcium ammonium nitrate to closely match the MS ratios. For sugarcane tissue culture plantlets, key indicators of medium suitability include leaf greenness, plantlets proliferation rate, and root number and length. In tissue culture, proliferation rate, root growth, leaf color, superoxide dismutase (SOD) activity, nitrogen content, and soluble protein content are critical indicators for assessing plant vitality and determining whether the growth environment is conducive to plant development.

### 4.1. Effect of Proliferation Rate

In this experiment, increasing ammonium nitrogen and nitrate nitrogen within a certain range enhanced the proliferation rate of sugarcane plantlets. Morphological parameters were most significant at treatment levels 3 and 4. However, as the ratio of the two nitrogen forms increased further, the morphological parameters of the six sugarcane varieties declined to varying degrees. Similarly, several studies support these findings. Xu et al. [20] demonstrated that appropriately increasing the proportion of ammonium nitrogen promotes cherry tomato fruit development and improves quality. Tomatoes grow best under normal temperature hydroponic conditions with a total nitrogen level of 8 and a ratio of ammonium nitrogen to nitrate nitrogen of 3:1 [21]. Wang et al. [22] observed that the above-ground dry weight of Coix variety Wanyi 2 plantlets showed an initial increase followed by a decrease as the nitrogen concentration increased. These studies align with the effects observed in this experiment regarding the impact of different nitrogen forms on the growth of sugarcane plantlets. Recent studies have found that the combination of ammonium and nitrate in appropriate ratios can enhance multiple agronomic traits in plants [23].

### 4.2. Effects on Root Growth

As the primary organ for absorption and metabolism in sugarcane, the root system is intricately linked to the aboveground parts. Its growth condition not only directly impacts its capacity to absorb water and nutrients but also influences the growth and development of the aboveground portions of the sugarcane. Plants primarily absorb nutrients through their roots, making the ratio of different nitrogen forms crucial to root growth and development. Qian et al. [24] found that increased application of two nitrogen forms can significantly promote rice root elongation. Similarly, Lewis et al. [25] observed that under hydroponic conditions, the appropriate application of nitrate nitrogen and ammonium nitrogen increased the number of roots in wheat and corn. Furthermore, it was highlighted that as nitrogen supply increases, the number of plant lateral roots initially increases and then decreases [26]. In this experiment, increased application of ammonium nitrogen and nitrate nitrogen significantly promoted the rooting rate of sugarcane plantlets across six test varieties. However, continued application of these nitrogen forms resulted in a decrease in the number of hairy roots and the length of the root system. This finding aligns with previous research, indicating consistency with past results. Additionally, using nitrate nitrogen alone led to an increase in environmental nitrate content, adversely affecting plant growth [27].

From the perspective of absorption and assimilation costs, ammonium nitrogen has lower energy consumption because it can be directly incorporated into the carbon chain during nitrogen assimilation, improving nitrogen utilization. However, high concentrations of ammonium nitrogen may cause ammonium salt toxicity in plants. Therefore, the combined application of both nitrogen forms effectively meets the growth and development needs of plants. In this experiment, all varieties of sugarcane plantlets maintained high biomass under the nitrogen ratios of treatment 3 and treatment 4. This indicates that these specific nitrogen ratios effectively meet the growth requirements of sugarcane plantlets.

### 4.3. Effects of Two Forms of Nitrogen Accumulation on Chlorophyll Content

This study elucidates the effects of different nitrogen forms and levels on nitrogen accumulation in plants. With increasing ammonium nitrogen and nitrate nitrogen treatments, the nitrogen content in sugarcane plantlets significantly increased in both forms. Most tested sugarcane original plantlets achieved the highest absorption values under treatments 3 and 4, with no significant difference between these two treatments, yet these values were significantly higher than those in other treatments. Nitrogen is a vital component of chlorophyll, and the chlorophyll content exhibits a very strong positive correlation with the net photosynthetic rate, with a correlation coefficient *R*^2^ as high as 0.97 [28]. Thus, nitrogen indirectly influences photosynthesis by affecting chlorophyll content. Research indicates that under non-stress conditions, plants primarily absorb nitrogen in the forms of ammonium salts and nitrates. The form of available nitrogen plays a critical role in plant photosynthesis and growth [29].

Globally, nitrogen assimilation costs account for 25% of the energy supplied by photosynthesis, and nitrogen consumption reduces the photosynthetic capacity of plants. Moreover, the form of nitrogen significantly impacts plant photosynthesis [30]. Some researchers suggest that the assimilation of ammonium nitrogen and nitrate nitrogen may consume NADPH through different pathways, resulting in variations in photosynthesis [31].

Although this study did not directly measure the photosynthesis of the original plantlets of the six sugarcane varieties, the trend of chlorophyll content was consistent with the changes in the two nitrogen forms. As the ratio of the two nitrogen fertilizers increased, chlorophyll content initially increased, reaching its peak under treatments 3 and 4, before subsequently decreasing. It was found that the excessive application of ammonium nitrogen or nitrate nitrogen reduces the chlorophyll content of tomato plantlets, thereby affecting photosynthesis and directly impacting plant biomass accumulation. Consequently, this trend is reflected in the combination of proliferation rate and rooting [32].

### 4.4. Effects of Two Forms of Nitrogen Accumulation on Soluble Protein Content

The present data revealed that, with the increase of ammonium nitrogen and nitrate nitrogen, the soluble protein content of the six tested varieties of sugarcane plantlets showed an increasing trend to varying degrees. The soluble protein content of each variety reached its peak in treatments 3 and 4. However, as the ratio of ammonium nitrogen to nitrate nitrogen continued to increase, the soluble protein content gradually decreased. This trend aligns with previous findings, which indicate that the combined application of two nitrogen forms, administered simultaneously in specific amounts, results in higher protein content compared to the use of a single nitrogen source [23]. For instance, in rice, the maximum activity of nitrogen assimilation enzymes, chlorophyll content, and photosynthetic activity were observed. Similarly, in wheat, an increase in total soluble protein content was noted, leading to the highest crop yield and nutritional value [33].

Soluble protein is the most active component among all protein constituents in plants, including various zymogens, enzyme molecules, and metabolic regulators. It plays a crucial role in physiological metabolism and reflects the nitrogen assimilation rate and nitrogen metabolism level of plants. The form of nitrogen primarily affects soluble protein content by influencing the nitrogen assimilation rate. Therefore, the changing trend in soluble protein content of the original plantlets of various sugarcane varieties in this experiment showed a high consistency with the accumulation of the two forms of nitrogen. This finding suggests that the form of nitrogen significantly affects the soluble protein content in plants.

Overall, the results indicate that increasing the ratio of ammonium nitrogen to nitrate nitrogen led to similar trends in the content of both nitrogen forms across the six sugarcane varieties. Treatments 3 and 4 were found to be conducive to nitrogen absorption. This trend mirrors that of soluble protein content, suggesting that these treatments also facilitate nitrogen assimilation, which is crucial for protein synthesis.

### 4.5. Effects of Two Forms of Nitrogen Accumulation on the Activity of Superoxide Dismutase (SOD)

Superoxide dismutase (SOD) activity is a common indicator for studying plant stress physiology as its change pattern intuitively reflects plant growth and physiological conditions. In this study, the SOD activity of sugarcane plantlets showed an upward trend with increasing ammonium nitrogen and nitrate nitrogen content in the culture medium. The SOD activity peaked under treatments 3 and 4, at which point the rate of reactive oxygen species (ROS) clearance exceeded their production rate. However, as the amounts of ammonium nitrogen and nitrate nitrogen continued to increase, SOD activity slowly decreased. This caused the production of reactive oxygen species (ROS) to exceed their removal, leading to damage of cell membranes. Similar studies reported that the mixed application of ammonium nitrogen and nitrate nitrogen within an appropriate range increased the antioxidant enzyme activity of cotton plantlets, reducing damage from lipid peroxidation [34]. Additionally, it was found that when wheat plantlets were cultured under six different gradients of nitrogen levels, the antioxidant enzyme activity followed the same pattern across three varieties [35,36]. As the nitrogen concentration increased, antioxidant enzyme activity initially rose and then declined. These findings are consistent with the results of this experiment. Furthermore, changes in SOD activity and soluble protein levels, both types of functional proteins, showed similar patterns. Among the six tested sugarcane varieties, the original plantlets under treatments 3 and 4 exhibited optimal SOD activity, significantly outperforming other treatments. However, there were no significant differences in the physiological status of the plantlets between treatments 3 and 4. Therefore, considering the need to control costs in production practice, treatment 3 should be chosen for the cultivation of sugarcane tissue culture plantlets.

## 5. Conclusions

In summary, the study found that tissue culture plantlets of six sugarcane varieties thrived significantly with the optimized formulations of calcium ammonium nitrate and ammonium sulfate, particularly in treatments 3 and 4. These treatments not only enhanced plantlets growth but also presented a cost-effective alternative to traditional MS media, which has become prohibitively expensive due to recent nitrate restrictions in China. Treatment 3 emerged as the most economical option, suggesting its suitability for large-scale application in tissue culture industries. Overall, this research offers a viable solution to current challenges in sugarcane tissue culture production, promoting both efficiency and cost savings.

## Figures and Tables

**Table 1:** The concentration of ammonium sulfate and calcium ammonium nitrate were determined for each treatment. In contrast, the control (CK) was assessed for the concentration of both ammonium nitrate and potassium nitrate.

Ion Types (Excluding Organic Matter)	Ion Concentrations (mM) in Control and Each Treatment						
	CK	Treatment 1	Treatment 2	Treatment 3	Treatment 4	Treatment 5	Treatment 6
NO_3_^−^	39.28	19.69	29.54	39.38	49.225	59.07	68.915
NH_4_^+^	20.49	10.3	15.44	20.59	25.74	30.89	36.03
H_2_PO_4_^−^	1.25	1.25	1.25	1.25	1.25	1.25	1.25
K^+^	20.045	10.65	15.35	20.005	24.74	29.44	34.14
Ca^2+^	2.99	1.5	2.25	3	3.75	4.5	5.25
Cl^−^	5.98	3	4.5	6	7.5	9	10.5
SO_4_^2−^	1.73	1.73	1.73	1.73	1.73	1.73	1.73
Mg^2+^	1.5	1.5	1.5	1.5	1.5	1.5	1.5
Mn^2+^	0.1	0.1	0.1	0.1	0.1	0.1	0.1
Zn^2+^	0.03	0.03	0.03	0.03	0.03	0.03	0.03
Na^+^	0.2002	0.002	0.002	0.002	0.002	0.002	0.002
MoO_4_^2−^	0.001	0.001	0.001	0.001	0.001	0.001	0.001
BO_3_^3−^	0.1	0.1	0.1	0.1	0.1	0.1	0.1
I^+^	0.005	0.005	0.005	0.005	0.005	0.005	0.005
Cu^2+^	0.0001	0.0001	0.0001	0.0001	0.0001	0.0001	0.0001
Co^2+^	0.0001	0.0001	0.0001	0.0001	0.0001	0.0001	0.0001
Fe^2+^	0.1	0.1	0.1	0.1	0.1	0.1	0.1
EDTA^2−^	0.1	0.1	0.1	0.1	0.1	0.1	0.1

**Table 2:** This table provides the prices of the chemical reagents used in the original MS formula, as well as the total cost.

	Chemical Name	Specification (g)	Price (RMB)	Quantity Used (g)	Total Amount (RMB)
Macronutrients	Potassium nitrate (KNO_3_)	500	50	190	19
	Ammonium nitrate (NH_4_NO_3_)	500	50	165	16.5
	Magnesium sulfate (MgSO_4_·7H_2_O)	500	20	37	1.48
	Potassium phosphate (KH_2_PO_4_)	500	24	17	0.816
Calcium salt	Calcium chloride (CaCl_2_)	500	22	33.2	1.4608
Trace elements	Manganese sulfate (MnSO_4_·4H_2_O)	500	33	2230	147.18
	Manganese sulfate (MnSO_4_·4H_2_O)	500	21	2.23	0.09366
	Zinc sulfate (ZnSO_4_·7H_2_O)	500	26	0.86	0.04472
	Boric acid (H_3_BO_3_)	500	20	0.62	0.0248
	Potassium iodide (KI)	100	92	0.083	0.07636
	Sodium molybdate (Na_2_MoO_4_·2H_2_O)	500	238	0.025	0.0119
	Copper sulfate (CuSO_4_·5H_2_O)	500	36	0.002.5	0.0018
	Cobalt chloride (CoCl_2_·6H_2_O)	100	48	0.002.5	0.0012
Iron salts	Ferrous sulfate (FeSO_4_·7H_2_O)	500	40	2.78	0.2224
	Sodium ethylenediaminetetraacetate (Na_2_-EDTA)	500	72	3.73	0.53712
Organic components	Glycine	100	13	0.2	0.026
	Vitamin B1 (thiamine)	100	62	0.05	0.031
	Vitamin B6	50	60	0.1	0.12
	Vitamin B3	50	55	0.05	0.055
	Inositol	100	40	10	4
	Agar	1000	210	800	168
	Sucrose	500	15	3000	90
Total					449.7

**Table 3:** The cost of preparing 100 L of medium using different methods.

Original MS Medium Cost (¥/yuan)	Cost of Formula 3 (¥/yuan)	Cost of Formula 4 (¥/yuan)	Commercial Powder Solid MS (¥/yuan)	Commercial Liquid MS (¥/yuan)
449.7	423.14	425.37	712.04	1503

## Data Availability

Data are included in the article.
